# Identification of two new protective pre-erythrocytic malaria vaccine antigen candidates

**DOI:** 10.1186/1475-2875-10-65

**Published:** 2011-03-16

**Authors:** Keith Limbach, Joao Aguiar, Kalpana Gowda, Noelle Patterson, Esteban Abot, Martha Sedegah, John Sacci, Thomas Richie

**Affiliations:** 1 U.S. Military Malaria Vaccine Program, Naval Medical Research Center, 503 Robert Grant Avenue, Silver Spring, MD, USA; 2 Henry M. Jackson Foundation for the Advancement of Military Medicine, 1401 Rockville Pike (Suite 600), Rockville, MD, USA; 3 Department of Microbiology and Immunology, University of Maryland School of Medicine, Baltimore, MD, USA

## Abstract

**Background:**

Despite years of effort, a licensed malaria vaccine is not yet available. One of the obstacles facing the development of a malaria vaccine is the extensive heterogeneity of many of the current malaria vaccine antigens. To counteract this antigenic diversity, an effective malaria vaccine may need to elicit an immune response against multiple malaria antigens, thereby limiting the negative impact of variability in any one antigen. Since most of the malaria vaccine antigens that have been evaluated in people have not elicited a protective immune response, there is a need to identify additional protective antigens. In this study, the efficacy of three pre-erythrocytic stage malaria antigens was evaluated in a *Plasmodium yoelii*/mouse protection model.

**Methods:**

Mice were immunized with plasmid DNA and vaccinia virus vectors that expressed one, two or all three *P. yoelii *vaccine antigens. The immunized mice were challenged with 300 *P. yoelii *sporozoites and evaluated for subsequent infection.

**Results:**

Vaccines that expressed any one of the three antigens did not protect a high percentage of mice against a *P. yoelii *challenge. However, vaccines that expressed all three antigens protected a higher percentage of mice than a vaccine that expressed PyCSP, the most efficacious malaria vaccine antigen. Dissection of the multi-antigen vaccine indicated that protection was primarily associated with two of the three *P. yoelii *antigens. The protection elicited by a vaccine expressing these two antigens exceeded the sum of the protection elicited by the single antigen vaccines, suggesting a potential synergistic interaction.

**Conclusions:**

This work identifies two promising malaria vaccine antigen candidates and suggests that a multi-antigen vaccine may be more efficacious than a single antigen vaccine.

## Background

Malaria kills approximately 863,000 people every year [1]. Although a variety of anti-malarial drugs exist, the cost of these drugs can be prohibitive in the relatively poor areas of the world where malaria is endemic. The wide-spread use of the most commonly employed drugs has also resulted in the expansion of drug-resistant parasites, rendering many of these drugs ineffective [2]. In the absence of inexpensive, highly potent drugs, vaccination represents the most cost-effective way of supplementing traditional malaria interventions.

A successful malaria vaccine will need to protect people against a large population of antigenically diverse malaria parasites. A vaccine based on a single isolate of a single antigen may not be able to elicit an immune response that is broad enough to protect individuals against this heterogeneous population. Therefore, an efficacious malaria vaccine may need to induce an immune response against multiple malaria antigens, a belief that has propelled the development of whole-organism malaria vaccines, such as the irradiated sporozoite vaccine and the genetically attenuated sporozoite vaccine [3,4].

A variety of malaria vaccine candidates are being evaluated in clinical trials throughout the world. The most advanced vaccine candidate, RTS,S, is currently being evaluated in a phase 3 trial at 11 sites in seven African countries. RTS,S is a recombinant protein vaccine based on the *Plasmodium falciparum *circumsporozoite protein (CSP). It has protected malaria-naïve adults against an experimental *P. falciparum *challenge and reduced malaria-associated episodes in children living in malaria endemic areas [5,6]. The level and duration of immunity induced by RTS,S, however, is relatively modest.

One way to potentially enhance the efficacy of RTS,S, or any other subunit malaria vaccine, would be to incorporate additional malaria antigens into the vaccine, thereby broadening the immune response elicited by the vaccine. At least one other malaria antigen has protected volunteers against a malaria challenge. A prime-boost regimen with adenovirus and poxvirus vectors expressing *P. falciparum *thrombospondin-related adhesive protein (TRAP) has protected volunteers against an experimental *P. falciparum *challenge [7]. A prime-boost regimen with DNA and adenovirus vectors expressing CSP and apical membrane antigen 1 (AMA1) has also protected volunteers against an experimental *P. falciparum *challenge [8]. Although the data from both of these clinical trials are not yet published, these studies indicate that CSP, TRAP and possibly AMA1 can induce protective immune responses in people. Unfortunately, most of the other malaria vaccine antigens evaluated in people have not induced significant levels of protection. For example, recombinant protein vaccines containing the C-terminal end of the merozoite surface protein 1 (MSP1_42_), the AMA1 ectodomain or a combination of three *P. falciparum *antigens (MSP1, MSP2 and ring-infected erythrocyte surface antigen (RESA)) have not induced significant levels of protection against natural infection in children living in malaria endemic regions [9-11]. Each of these vaccines, however, may have induced some level of strain-specific protection against the *P. falciparum *strain from which the vaccine antigen was derived [11,12]. Since an immune response against multiple malaria antigens may be necessary to protect a high percentage of people against the large number of antigenically diverse *P. falciparum *strains throughout the world, there is a great need to identify new malaria vaccine antigens.

In this report, the efficacy of three malaria vaccine antigens was evaluated in a *P. yoelii*/mouse model. Although these three pre-erythrocytic stage antigens, PY03011, PY03424 and PY03661, were independently identified by our bioinformatic and genomic analyses, two of the antigens (or their orthologs) were previously described (PY03011 = PyUIS3, PY03424 = falstatin) [13,14]. Protection studies with DNA and vaccinia virus vaccine vectors expressing these antigens suggest that two of the antigens, PY03011 and PY03424, can protect mice against a *P. yoelii *sporozoite challenge.

## Methods

### Down-selection of vaccine candidate genes

*P. falciparum *and *P. yoelii *express approximately 5,800 genes. It is not feasible to evaluate the vaccine potential of that many genes. Therefore, various methods were used to down-select the most promising vaccine candidates. Assuming that a vaccine based on a pre-erythrocytic antigen is more likely to be successful than a vaccine based on an erythrocytic antigen, the down-selection process focused on sporozoite and liver stage antigens. To identify promising sporozoite antigens, genomic and proteomic information contained in pre-existing malaria databases was evaluated [15,16]. To identify promising liver stage antigens, an expression library created with material isolated from *P. yoelii*-infected liver cells was evaluated [17]. The *P. falciparum *genes encoding the down-selected sporozoite and liver stage antigens were cloned using a high-throughput cloning strategy [18]. Evaluation of the proteins encoded by these genes with antisera from volunteers who had received a *P. falciparum *irradiated sporozoite vaccine identified 20 promising vaccine candidates [19].

### Generation of DNA and vaccinia virus vaccine vectors

The re-annotated single exon PY03011 gene was isolated from *P. yoelii *(17XNL) genomic DNA by PCR with the primers, 5'-TGGATCCATGAAAGTGTATAAAATGAACACTCTC-3' and 5'-TGGATCCTCATTTTGGTTGATATTGTTCTTTAAG-3'. The DNA-PY03011 vaccine vector was generated by cloning the PY03011 gene from this PCR reaction into the *Bam*HI site of the DNA vaccine vector, VR1020 (Vical Inc., San Diego, CA). This cloning reaction positions the full length PY03011 gene downstream from a cytomegalovirus (CMV) immediate-early (IE) promoter and in-frame with a human tissue plasminogen activator (TPA) signal sequence. Since the PY03011 protein contains a signal sequence, cloning the PY03011 gene into VR1020 downstream from an in-frame TPA signal sequence results in a PY03011 construct that contains two signal sequences. The vaccinia-PY03011 vaccine vector was generated using a host range selection system [20]. The full length PY03011 gene in this vector is inserted into the vaccinia virus A-type inclusion body (ATI) locus and is under the transcriptional control of a synthetic early/late (E/L) promoter [21].

Exon 2 of the re-annotated PY03424 gene was isolated from *P. yoelii *(17XNL) genomic DNA by PCR with the primers, 5'-TGGATCCTACTCTTTTGACATTGTAAACGAG-3' and 5'-TGGATCCTTATTGGACAGTTACGTATAAAATTTTAG-3'. The DNA-PY03424 vaccine vector and the vaccinia-PY03424 vaccine vector were generated with the same reagents and techniques used to generate the DNA-PY03011 and vaccinia-PY03011 vectors. The DNA and vaccinia vaccine vectors expressing PY03424 (exon 2) do not contain the first 26 codons of the PY03424 gene. Since the first 21 codons of the PY03424 gene encode a signal sequence, the PY03424 proteins expressed by the DNA-PY03424 and vaccinia-PY03424 vectors do not contain the native PY03424 signal sequence. To enhance expression, the DNA-PY03424 vector was engineered to express a PY03424 protein with a TPA signal sequence and the vaccinia-PY03424 vector was engineered to express a PY03424 protein with a human decay accelerating factor (DAF) signal sequence.

The PY03661 gene was isolated from *P. yoelii *(17XNL) genomic DNA by PCR with the primers, 5'-TGGATCCATGTTTCGATCTGATTCCCATTTCC-3' and 5'-TGGATCCTTATGTTTGATGATAATTTTCTTTCG-3'. The DNA-PY03661 vaccine vector and the vaccinia-PY03661 vaccine vector were generated with the same reagents and techniques used to generate the other DNA-*P. yoelii *and vaccinia-*P. yoelii *vectors. Since the native PY03661 gene does not contain a signal sequence, the vaccinia-PY03661 expression cassette was constructed with a DAF signal sequence. Therefore, the DNA-PY03661 vector expresses a PY03661 protein with a TPA signal sequence and the vaccinia-PY03661 vector expresses a PY03661 protein with a DAF signal sequence.

The DNA-*P. yoelii *vaccines were manufactured to pre-clinical grade specifications by Puresyn, Inc. (Malvern, PA). The vaccinia-*P. yoelii *vaccines were propagated in RK-13 cells (rabbit kidney cells; ATCC CCL37) using standard laboratory procedures [22].

### Mice and parasites

Female CD1 outbred mice (5-6 weeks old) were purchased from Charles River Laboratories (Wilmington, MA). *P. yoelii *(17XNL non-lethal strain) parasites were maintained by alternating passage in *Anopheles stephensi *mosquitoes and female CD1 outbred mice.

### Production of recombinant *P. yoelii *proteins

PY03011, PY03424 (exon 2) and PY03661 recombinant proteins were generated with a wheat germ cell-free expression system [23]. In brief, *P. yoelii *gene-specific RNA was generated from a plasmid containing an SP6-promoted *P. yoelii*-glutathione S-transferase (GST) tagged construct with SP6 RNA polymerase. The *P. yoelii *RNA transcripts were translated into recombinant *P. yoelii *protein in a wheat germ cell-free extract (CellFree Sciences Co., Yokohama, Japan). The GST-tagged *P. yoelii *proteins were affinity purified with a glutathione sepharose resin and cleaved from the GST tag with tobacco etch virus protease (Invitrogen Corp., Carlsbad, CA).

### Generation of *P. yoelii *protein-specific antisera with recombinant *P. yoelii *proteins

Female CD1 mice were injected subcutaneously in the tail and scruff of the neck on days 0 and 29 with 10 μg of PY03661, PY03424 (exon 2) or PY03661 recombinant protein adjuvanted in Montanide™ ISA720. On day 38, the mice were bled and *P. yoelii *protein-specific antisera prepared.

### Indirect fluorescent antibody analyses

Indirect fluorescent antibody (IFA) assays with sporozoite and blood stage parasites were performed as previously described [24]. In brief, serial dilutions of *P. yoelii *protein-specific antisera were incubated with *P. yoelii *(17XNL) sporozoites or blood from *P. yoelii*-infected mice. Parasites were visualized with a fluorescein isothiocyanate (FITC) conjugated goat anti-mouse IgG (KPL Inc., Gaithersburg, MD). IFA analyses with liver stage parasites were performed as previously described [25]. In brief, mice were infected with *P. yoelii *sporozoites and livers were harvested 48 hours post-infection. *P. yoelii*-infected liver sections were prepared and incubated with *P. yoelii *protein-specific antisera. Parasites were visualized with a FITC conjugated goat anti-mouse IgG. Evans blue (0.02%) counterstain was added to the secondary antibody, providing a red background to contrast the green FITC fluorescence when excited at the same wavelength.

### *In vitro *expression analyses

Protein expression from the DNA and vaccinia vectors was evaluated by Western blot analyses. DNA-*P. yoelii *plasmids were transfected into RK-13 cells with lipofectamine 2000CD (Invitrogen Corp., Carlsbad, CA). Vaccinia-*P. yoelii *infections were performed in RK-13 cells. Cell lysates were run on 4-20% Tris-Glycine acrylamide gels (Invitrogen Corp., Carlsbad, CA), transferred to Immobilon-P polyvinylidene difluoride (PVDF) membranes (Millipore Corp., Bedford, MA) and probed with antisera from mice immunized with PY03011, PY03424 or PY03661 vaccines. Proteins were detected with an alkaline phosphatase Western-Light Chemiluminescent Detection System (Tropix Inc., Bedford, MA) and an alkaline phosphatase colorimetric substrate (KPL Inc., Gaithersburg, MD).

### Protection studies

Female CD1 mice were injected intramuscularly in the tibialis anterior muscle with 100 μl of vaccine (50 μl in each leg) using a 0.3 ml syringe and a 29G1/2 needle (Becton Dickinson Co., Franklin Lakes, NJ) fitted with a plastic collar cut from a micropipette tip [26]. The DNA vaccine vectors were prepared in 1X Phosphate Buffered Saline (PBS) and diluted to the appropriate concentration for vaccination in 1X PBS. The vaccinia vaccine vectors were prepared in 1 mM Tris (9.0) and diluted to the appropriate concentration for vaccination in 1X PBS. Mice were challenged intravenously in the tail vein with 300 *P. yoelii *(17XNL) sporozoites using a 1 ml syringe and 26G1/2 needle (Becton Dickinson Co., Franklin Lakes, NJ). Sporozoites were hand dissected from infected mosquito salivary glands and diluted for challenge in M199 medium containing 5% normal mouse serum (Gemini Bio-Products, West Sacramento, CA).

In protection study 1, 14 mice per group were primed on day 0 with 100 μg of the appropriate DNA-*P. yoelii *vaccine vector and boosted on day 40 with 5 × 10^7 ^plaque forming units (pfu) of the corresponding vaccinia-*P. yoelii *vaccine vector. Mice immunized with a combination of vectors expressing PY03011, PY03424 and PY03661 were primed with a total of 300 μg of the DNA-*P. yoelii *vectors and boosted with a total of 1.5 × 10^8 ^pfu of the vaccinia-*P. yoelii *vectors. Vaccine vectors expressing PyCSP were included in each study as a positive control. On day 50, the mice were bled and sera prepared. On day 54, the mice were challenged with 300 *P. yoelii *sporozoites. On days 61-68, parasitaemia was evaluated by examining Giemsa-stained blood smears. Mice were considered positive if parasites were observed in any sample. To gauge the severity of the challenge, four groups of naïve CD1 mice were challenged with four suboptimal doses of *P. yoelii *sporozoites (calculated through serial dilution to be approximately 100, 33.3, 11.1 or 3.7 sporozoites per mouse). From these infectivity control mice, an ID_50 _was calculated. (An ID_50_, or infectious dose 50, equals the dose of sporozoites required to infect 50% of the mice.) Extrapolation from these results indicated that the mice injected with 300 *P. yoelii *sporozoites were challenged with a dose equivalent to seven times the ID_50 _dose.

In protection study 2, 14 mice per group were primed on day 0 with 100 μg of the appropriate DNA-*P. yoelii *vaccine vector and 30 μg of a DNA vector expressing murine granulocyte-macrophage colony-stimulating factor (mGM-CSF) and boosted on day 42 with 3.3 × 10^7 ^pfu of the corresponding vaccinia-*P. yoelii *vaccine vector. Mice immunized with two or three DNA-*P. yoelii *vectors were primed with a total of 200 μg or 300 μg of the DNA-*P. yoelii *vectors and 30 μg of the DNA-mGM-CSF vector and boosted with a total of 6.6 × 10^7 ^pfu or 1 × 10^8 ^pfu of the vaccinia-*P. yoelii *vectors. Three separate groups of negative control mice were immunized with three different doses of DNA and vaccinia vectors that do not express a *P. yoelii *antigen. One group was primed with 100 μg of an "empty" DNA vector and 30 μg of a DNA-mGM-CSF vector and boosted with 3.3 × 10^7 ^pfu of an "empty" vaccinia vector. A second group was primed with 200 μg of an "empty" DNA vector and 30 μg of a DNA-mGM-CSF vector and boosted with 6.6 × 10^7 ^pfu of an "empty" vaccinia vector. A third group was primed with 300 μg of an "empty" DNA vector and 30 μg of a DNA-mGM-CSF vector and boosted with 1 × 10^8 ^pfu of an "empty" vaccinia vector. On day 52, the mice were bled and sera prepared. On day 57, the mice were challenged with 300 *P. yoelii *sporozoites. On days 64-71, parasitaemia was evaluated by examining Giemsa-stained blood smears. Mice were considered positive if parasites were observed in any sample. To gauge the severity of the challenge, four groups of naive mice were challenged with four suboptimal doses of *P. yoelii *sporozoites (100, 33.3, 11.1 or 3.7 sporozoites). From these infectivity control mice, it was calculated that the mice injected with 300 *P. yoelii *sporozoites in this study were challenged with a dose equivalent to 13.6 times the ID_50 _dose.

The regimens for the two protection studies were slightly different. For example, the dose of the individual vaccinia-*P. yoelii *vectors was slightly higher in protection study 1 (5 × 10^7 ^pfu) than protection study 2 (3.3 × 10^7 ^pfu). Consequently, the total dose of the trivalent vaccine was 1.5 × 10^8 ^pfu in protection study 1 and 1 × 10^8 ^pfu in protection study 2. Additionally, in protection study 2, the DNA vectors were mixed with a DNA-mGM-CSF plasmid. Although previous studies had indicated that co-administration of a DNA-PyCSP vector with a DNA-mGM-CSF plasmid could enhance the immunogenicity and efficacy of a DNA-vaccinia prime-boost regimen [27], this enhancement is greater in inbred mouse strains (BALB/c and C57BL/6) than outbred strains [28]. Therefore, it is not surprising that the DNA-mGM-CSF plasmid did not appear to enhance the efficacy of the PyCSP or trivalent *P. yoelii *vaccines in protection study 2, relative to protection study 1.

### Statistical analyses

Protection results were analyzed by a Fisher's Exact Test with GraphPad Prism 5.03 software (GraphPad Software Inc., LaJolla, CA).

## Results

### Genomic characterization of three *P. yoelii *vaccine antigens

Analysis of pre-erythrocytic *P. falciparum *proteins with sera from human volunteers immunized with a *P. falciparum *irradiated sporozoite vaccine identified 20 promising vaccine antigens [19]. To evaluate the vaccine potential of these proteins in a murine protection model, vaccine vectors that express the *P. yoelii *ortholog of three of these antigens, PY03011, PY03424 and PY03661, were generated.

### PY03011

PY03011 is predicted by PlasmoDB [29], a *Plasmodium *database, to be the ortholog of the *P. falciparum *gene, PF13_0012. PF13_0012 is predicted by PlasmoDB to be a single exon gene that encodes a protein that is 229 amino acids long. PY03011 is predicted by PlasmoDB to contain two exons and encode a protein that is 241 amino acids long. The first exon is predicted to encode the first 16 amino acids and the second exon is predicted to encode the remaining 225 amino acids. A previous study, however, annotated the PY03011 gene to be a single exon gene that encodes a protein that is 220 amino acids long [30]. The re-annotated PY03011 protein is more homologous to PF13_0012 than the PlasmoDB-annotated PY03011 protein (30% vs. 28%). The single exon annotation is also consistent with the annotations of the *P. falciparum*, *Plasmodium berghei*, *Plasmodium chabaudi*, *Plasmodium knowlesi *and *Plasmodium vivax *orthologs of this gene, which are predicted by PlasmoDB to be single exon genes. Based upon these data, the studies in this report were performed with a single exon PY03011 gene that encodes a protein that is 220 amino acids long [30].

**Figure 1:**
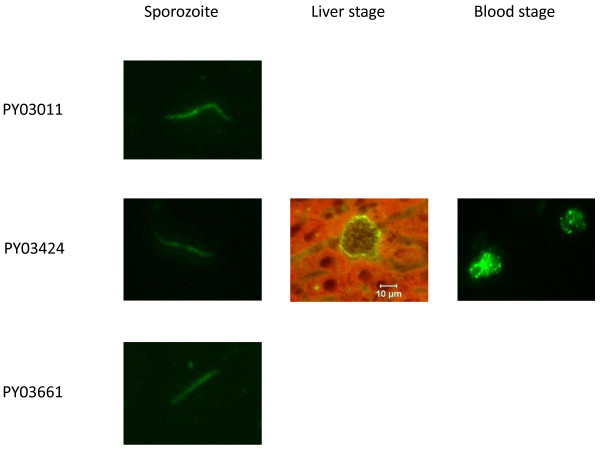
**Stage-specific expression of PY03011, PY03424 and PY03661**. *P. yoelii *sporozoite, liver stage and blood stage IFA slides were analyzed with antigen-specific antisera from mice immunized with PY03011, PY03424 or PY03661 recombinant proteins. Liver stage sections were prepared from tissue harvested 48 hours after infection with *P. yoelii *sporozoites. Results are not shown if expression was not detected.

The re-annotated PY03011 protein is predicted to contain a signal sequence with a cleavage site between amino acids 30-31 and a transmembrane domain between amino acids 59-81. IFA analyses with PY03011-specific antisera indicate that PY03011 is expressed in the sporozoite, but not in the liver or blood stages of the *P. yoelii *life-cycle (Figure 1). A previous study indicated that this protein was expressed in the sporozoite and liver stages [13]. Therefore, it is likely that PY03011 is expressed in the liver, but at levels that are below the level of detection with the serological reagents used in the present study. The genetic characteristics of the re-annotated PY03011 gene are summarized in Table 1.

**Table 1:** Genetic characteristics of 3 *P. yoelii *vaccine antigens

Gene	Size	Exons	Signal/TM	Homology (Py vs. Pf)	Expression (Stage)
PY03011	220 a.a.	1	Yes/Yes	30%	S/(L)
PY03424	357 a.a	2	Yes/No	33%	S/L/B
PY03661	225 a.a.	1	No/No	60%	S/(L)

The genetic structure, homology and stage-specific expression of the re-annotated PY03011 gene, the re-annotated PY03424 gene and the PlasmoDB-annotated PY03661 gene are listed. Homology (Py vs. Pf) represents the amino acid homology between the *P. yoelii *and *P. falciparum *orthologs. Expression (stage) represents the stage-specific expression of the *P. yoelii *proteins. Previous studies indicated that PY03011/PyUIS3 and the *P. falciparum *PY03661 ortholog, PFC0555c, are expressed in the liver [13,19]. Although we did not detect expression of PY03011 and PY03661 in the liver, it is likely that both proteins are expressed in the liver at levels that are below the level of detection with the serological reagents used in this study. To indicate this possibility, liver stage expression of PY03011 and PY03661 is presented in parentheses. Abbreviations: a.a. = amino acids, signal = signal sequence, TM = transmembrane region, Py = *P. yoelii*, Pf = *P. falciparum*, S = sporozoite, L = liver stage, B = blood stage.

### PY03424

PY03424 is predicted by PlasmoDB to be the ortholog of the *P. falciparum *gene, PFI0580c. PFI0580c is predicted by PlasmoDB to contain two exons and encode a protein that is 413 amino acids long. The first exon is predicted to encode the first 22 amino acids and the second exon is predicted to encode the remaining 391 amino acids. PY03424 is predicted by PlasmoDB to contain two exons and encode a protein that is 1,856 amino acids long. The first exon is predicted to encode the first 1,521 amino acids and the second exon is predicted to encode the remaining 335 amino acids. We believe that PY03424 is not annotated correctly and have re-annotated this gene. The re-annotated PY03424 gene is predicted to contain two exons and encode a protein that is 357 amino acids long. The first exon of the re-annotated gene encodes 22 amino acids and the second exon encodes the remaining 335 amino acids. The re-annotated PY03424 protein is significantly more homologous to PFI0580c than the PlasmoDB-annotated PY03424 protein (33% vs. 6%). A comparison of PY03424 with other *Plasmodium *orthologs also suggests that the PlasmoDB annotation is not correct.

The re-annotated PY03424 protein is predicted to contain a signal sequence with a cleavage site between amino acids 21-22. IFA analyses with PY03424-specific antisera indicate that PY03424 is expressed in sporozoites, on the parasitophorous vacuole of the liver stage and in the blood stage (Figure 1). This profile is similar to the expression profile of the *P. falciparum *PFI0580c ortholog, which is expressed in the sporozoite, liver and blood stages of the *P. falciparum *life-cycle [14,15,19]. The genetic characteristics of the re-annotated PY03424 gene are summarized in Table 1.

### PY03661

PY03661 is predicted by PlasmoDB to be the ortholog of the *P. falciparum *gene, PFC0555c. PFC0555c is predicted by PlasmoDB to be a single exon gene and encode a protein that is 233 amino acids long. PY03661 is predicted to be a single exon gene and encode a protein that is 225 amino acids long. The homology between the PFC0555c and PY03661 proteins is 60%. PY03661 does not appear to contain a signal sequence or a transmembrane domain. IFA analyses with PY03661-specific antisera indicate that PY03661 is expressed in sporozoites, but not in the liver or blood stages (Figure 1). The *P. falciparum *PFC0555c ortholog is expressed in the sporozoite and liver stages of the *P. falciparum *life-cycle [19]. Since PFC0555c is expressed in the liver stage, it is likely that PY03661 is also expressed in the liver, but at levels that are below the level of detection with the serological reagents used in this study. The genetic characteristics of PY03661 are summarized in Table 1.

### Construction and analyses of DNA and vaccinia virus vaccine vectors expressing PY03011, PY03424 or PY03661

**Figure 2:**
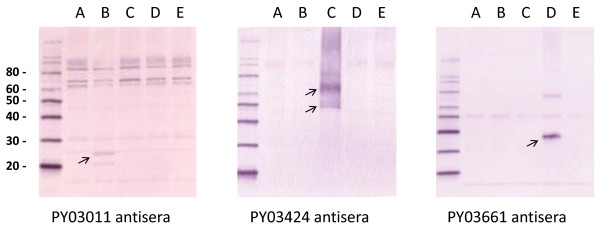
**Expression of *P. yoelii *proteins from DNA-*P. yoelii *vectors**. RK-13 cells were transfected with an "empty" DNA vaccine vector (lane A) or DNA vaccine vectors that express PY03011 (lane B), PY03424 (lane C), PY03661 (lane D) or PyCSP (lane E). Lysates were run on an acrylamide gel, transferred to a PVDF membrane and probed with antisera from mice immunized with DNA and vaccinia vectors that express PY03011, PY03424 or PY03661. Arrows indicate the major *P. yoelii *protein products. Molecular weight markers (with kilodaltons designations) are shown in the first lane.

**Figure 3:**
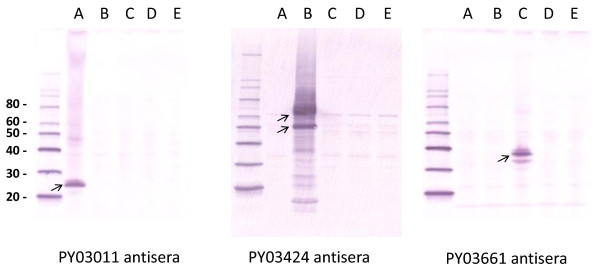
**Expression of *P. yoelii *proteins from vaccinia-*P. yoelii *vectors**. RK-13 cells were infected with vaccinia vectors that express PY03011 (lane A), PY03424 (lane B), PY03661 (lane C) or PyCSP (lane D), or an "empty" vaccinia vector (lane E). Lysates were run on an acrylamide gel, transferred to a PVDF membrane and probed with antisera from mice immunized with PY03011, PY03424 or PY03661 recombinant proteins. Arrows indicate the major *P. yoelii *protein products. Molecular weight markers (with kilodaltons designations) are shown in the first lane.

DNA and vaccinia virus vaccine vectors expressing the re-annotated PY03011 gene, the second exon of the re-annotated PY03424 gene or the PY03661 gene were generated. Western blot analyses with antigen-specific antisera indicate that the DNA (Figure 2) and vaccinia virus (Figure 3) vectors express the appropriate *P. yoelii *protein.

### Protection studies in *P. yoelii*/mouse model

**Figure 4:**
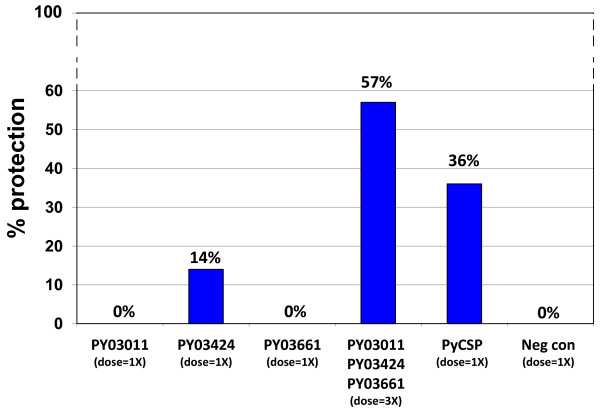
**Protection study 1**. Fourteen CD1 outbred mice per group were immunized in a prime-boost regimen with DNA and vaccinia vectors that express PY03011, PY03424 or PY03661, or a combination of vectors that express all three *P. yoelii *antigens. Positive control mice were immunized with DNA and vaccinia vectors that express PyCSP. Negative control mice were immunized with DNA and vaccinia vectors that do not express a *P. yoelii *antigen. Groups are designated (dose = 1X) or (dose = 3X) to represent the relative quantity of the DNA and vaccinia vectors that they received. The mice were challenged with 300 *P. yoelii *sporozoites and evaluated for parasitaemia by examining Giemsa-stained blood smears.

To evaluate the vaccine potential of the three *P. yoelii *antigens, CD1 outbred mice were immunized in a heterologous prime-boost regimen with DNA and vaccinia virus vectors that express PY03011, PY03424 or PY03661, or a combination of these vectors (Table 2). As a positive control, mice were immunized with DNA and vaccinia vectors that express *P. yoelii *CSP (PyCSP). As a negative control, mice were immunized with "empty" DNA and vaccinia vectors that do not express a *P. yoelii *protein. Two weeks after the vaccinia vector boost, the mice were challenged with 300 *P. yoelii *sporozoites. Seven through fourteen days after the challenge, protection against blood stage parasitaemia was evaluated by examining Giemsa-stained blood smears. None of the 14 mice immunized with vectors that express PY03011 or PY03661 were sterilely protected (0% protection) and only two of 14 mice immunized with vectors that express PY03424 were sterilely protected (14% protection). However, eight of 14 mice immunized with all three antigens were sterilely protected (57% protection) (Figure 4). The protection elicited by these three *P. yoelii *antigens was greater than the protection elicited by PyCSP (57% vs. 36%).

**Table 2:** Regimens for protection studies

Protection study 1:			
Prime ← (6 wk) →	Boost ← (2 wk) →	Challenge ← (1 wk) →	Monitor parasitaemia
Day 0	Day 40	Day 54	Days 61-68
DNA vectors (100 ug/vector)	Vaccinia vectors (5 × 10^7 ^pfu/vector)	300 Py spz	Blood smears
			
**Protection study 2:**			
**Prime ← (6 wk) →**	**Boost ← (2 wk) →**	**Challenge ← (1 wk) →**	**Monitor parasitaemia**

Day 0	Day 42	Day 57	Days 64-71
DNA vectors (100 ug/vector)	Vaccinia vectors (3.3 × 10^7 ^pfu/vector)	300 Py spz	Blood smears
DNA-mGM-CSF (30 ug/vector)			

**Figure 5:**
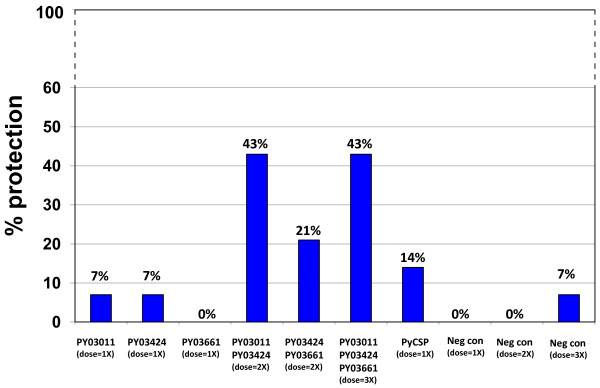
**Protection study 2**. Fourteen CD1 outbred mice per group were immunized in a prime-boost regimen with DNA and vaccinia vectors that express PY03011, PY03424 or PY03661, or a combination of vectors that express two or all three *P. yoelii *antigens. Positive control mice were immunized with DNA and vaccinia vectors that express PyCSP. Three separate groups of negative control mice were immunized with three different doses of DNA and vaccinia vectors that do not express *P. yoelii *antigens. Groups are designated (dose = 1X), (dose = 2X) or (dose = 3X) to represent the relative quantity of the DNA and vaccinia vectors that they received. The mice were challenged with 300 *P. yoelii *sporozoites and evaluated for parasitaemia by examining Giemsa-stained blood smears.

To confirm these results and determine which combination of antigens was responsible for protection, a second efficacy study was performed. CD1 outbred mice were immunized with DNA and vaccinia virus vectors that express PY03011, PY03424 or PY03661 (Table 2). In this study, however, separate groups of mice were immunized with a combination of vectors that express PY03011 and PY03424, or PY03424 and PY03661, or all three *P. yoelii *antigens. The PY03011 and PY03661 combination was not tested since previous studies had suggested that PY03424 was the primary protective antigen. As a positive control, mice were immunized with DNA and vaccinia vectors that express PyCSP. Since the mice immunized with multiple vectors received two or three times more vaccine than the mice immunized with a single vector, three separate groups of negative control mice were immunized with the same amount of "empty" DNA and vaccinia vectors as the mice that received either one, two or three vaccine vectors. Two weeks after the vaccinia vector boost, the mice were challenged with 300 *P. yoelii *sporozoites. Seven through fourteen days after the challenge, protection against blood stage parasitaemia was evaluated by examining Giemsa-stained blood smears. None of the mice immunized with vectors that express PY03661 were protected (0% protection) and only one of 14 mice immunized with vectors that express PY03011 or PY03424 were protected (7% protection). However, six of 14 mice immunized with PY03011 and PY03424 were protected (43% protection), three of 14 mice immunized with PY03424 and PY03661 were protected (21% protection) and six of 14 mice immunized with all three *P. yoelii *antigens were protected (43% protection) (Figure 5). The protection elicited by PY03011 and PY03424 is statistically significant (PY03011/PY03424 (dose = 2X) vs. Neg con (dose = 2X), p = 0.0159). Similar to the previous study, the protection elicited by the combination of PY03011 and PY03424 or all three antigens was greater than the protection elicited by PyCSP (43% vs. 14%).

## Discussion

In this report, the efficacy of three pre-erythrocytic stage malaria antigens, PY03011, PY03424 and PY03661, was evaluated. DNA and vaccinia virus vectors expressing these three antigens were evaluated in two *P. yoelii *protection studies. In the first study, a trivalent vaccine that expressed all three antigens protected a significantly higher percentage of mice than vaccines that expressed either antigen alone. Since the percentage of mice protected by the trivalent vaccine (57%) exceeded the sum of the percentages protected by the univalent vaccines (14%), these results suggest a potential synergistic interaction. In the second study, a bivalent vaccine that expressed PY03011 and PY03424 protected an equivalent percentage of mice as the trivalent vaccine, suggesting that PY03011 and PY03424 are the primary antigens responsible for protection. A bivalent vaccine that expressed PY03424 and PY03661 also protected a higher percentage of mice than either vaccine alone. However, the level of protection induced by this bivalent vaccine was not statistically significant relative to the PY03424, PY03661 or negative control groups. Similar to the first study, the number of mice protected by the trivalent vaccine (43%) or the PY03011 and PY03424 bivalent vaccine (43%) was larger than the sum protected by the univalent vaccines (14%), a result consistent with synergistic protection. These studies indicate that PY03011 and PY03424, and their *P. falciparum *orthologs, are potential malaria vaccine antigens.

PY03011 and PY03424, and/or their *P. falciparum *or *P. berghei *orthologs, have been previously characterized. PY03011, and its *P. berghei *ortholog, were initially identified by differential stage-specific expression studies, which resulted in it being designated PyUIS3 (upregulated in infectious sporozoites 3) [31,32]. Further studies indicated that PyUIS3/PY03011 is expressed in the liver stage parasitophorous vacuole, where it binds to the host cell fatty acid carrier, liver-fatty acid binding protein (L-FABP), and facilitates the importation of fatty acids from the hepatocyte to the parasite [13]. Down-regulation of L-FABP inhibits parasite growth. Therefore, although the parasite can synthesize fatty acids, it appears that it supplements this endogenous fatty acid production by importing fatty acids from the host [13]. PyUIS3/PY03011 is homologous with a family of *Plasmodium *proteins called early transcribed membrane proteins (ETRAMPs). All of the ETRAMPs share a similar structure; an N-terminal signal sequence followed by a short lysine-rich region, a second transmembrane domain and a C-terminal region of variable length [33]. Like PyUIS3/PY03011, ETRAMPs have been shown to localize to the parasitophorous vacuole. Unlike PyUIS3/PY03011, which is expressed in the sporozoite and liver stages, many of the ETRAMPs are expressed exclusively in the ring stage. ETRAMPs appear to localize to the liver stage or blood stage parasitophorous vacuole and have been shown to interact with host proteins. For example, PyUIS3/PY03011 interacts with L-FABP [13] and PFE1570w, another ETRAMP, interacts with human apolipoproteins [34]. Therefore, this family of proteins may interact with multiple host proteins.

A PyUIS3/PY03011-knockout parasite, Pyuis3(-), can develop into sporozoites and invade hepatocytes, but can not develop into merozoites, indicating that PyUIS3/PY03011 is not required for sporozoite development or sporozoite invasion of salivary glands or hepatocytes, but is required for liver stage development [35]. Mice immunized with Pyuis3(-) knockout parasites are protected against a wild-type *P. yoelii *challenge [35]. Therefore, an immune response against PyUIS3/PY03011 is not essential for protection. However, since PyUIS3/PY03011 is essential for hepatocyte development, it is not surprising that an immune response against this protein can help protect mice against a *P. yoelii *challenge.

PFI0580c, the *P. falciparum *ortholog of PY03424, encodes a putative cysteine protease inhibitor, falstatin [14]. This protein can inhibit the *P. falciparum *cysteine proteases, falcipain-2 and falcipain-3, as well as other *Plasmodium *and human cysteine proteases. Western and mass spectrophotometry analyses indicate that PFI0580c is expressed in sporozoites, as well as the ring, schizont and merozoite stages of the *P. falciparum *life-cycle, but not in trophozoites, the stage at which cysteine protease activity is greatest [14]. Antibodies against falstatin can inhibit merozoite infection of erythrocytes [14]. Therefore, this protein appears to be involved in erythrocyte invasion.

The *P. berghei *ortholog, *P. berghei *inhibitor of cysteine proteases (PbICP), has also been well characterized [36]. Similar to PY03424 and PFI0580c, PbICP is expressed in multiple stages of the parasite life-cycle. In sporozoites, it localizes to micronemes and is secreted by gliding sporozoites. In infected liver cells, it localizes to the parasitophorous vacuole. PbICP appears to play an important role in both of these stages. Pre-incubation of sporozoites with PbICP-specific antibody inhibits sporozoite infection of HepG2 cells. Therefore, this protein appears to play a role in sporozoite invasion of hepatocytes. In addition, HepG2 cells transfected with a plasmid expressing PbICP are resistant to apoptosis-inducing reagents. Therefore, PbICP may inhibit the programmed cell death of parasite-infected liver cells, perhaps by inhibiting one or more of the cellular proteases involved in this process. These studies indicate that the PY03424 orthologs, falstatin and PbICP, play a critical role in multiple stages of the parasite life-cycle, including sporozoite invasion of hepatocytes, liver stage development and merozoite infection of erythrocytes.

It is not known what the correlates of protection are in these studies. PyUIS3/PY03011 is expressed in the sporozoite and liver stages [13]. Since PyUIS3-knockout parasites can infect hepatocytes, this protein is not required for sporozoite infection of hepatocytes [35]. Therefore, antibodies against PY03011/PyUIS3 may not have an impact on sporozoite infectivity. Since PyUIS3-knockout parasites cannot develop into functional merozoites, this protein is essential for liver stage development [35]. PyUIS3 localizes to the liver stage parasitophorous vacuole and should not be accessible to circulating antibodies. Therefore, the protection induced by a PY03011-based vaccine may be more dependent on a cellular response than a humoral response.

The PY03424 orthologs, falstatin and PbICP, play critical roles in multiple stages of the parasite life-cycle, including sporozoite infection of hepatocytes, liver stage development and merozoite infection of erythrocytes. Antibodies against these proteins can inhibit sporozoite infection of hepatocytes and merozoite infection of erythrocytes [14,36]. Therefore, PY03424-specific antibodies may have played a critical role in the protection observed in this study. However, since this protein also appears to be involved in inhibiting apoptosis of infected hepatocytes, PY03424-specific T cell responses may have also played a role in protection.

The protection studies reported here were performed in CD1 outbred mice. Although studies with other malaria antigens have indicated that higher levels of protection can be attained in inbred mouse strains, protection is often antigen and strain-specific. For example, a DNA-PyCSP vaccine vector can protect BALB/c (H-2^d^) mice against a *P. yoelii *challenge, but cannot protect a high percentage of A/J (H-2^a^) or B10.BR (H-2^k^) mice. Conversely, a DNA-PyHEP17 vaccine vector can protect A/J and B10.BR mice, but cannot protect a high percentage of BALB/c mice [37]. To avoid the possibility of missing potentially protective vaccine antigens due to HLA-restricted responses, protection studies were performed in CD1 outbred mice.

These results suggest that combining vaccine antigens can have a synergistic impact on protection. Specifically, vaccine combinations with vectors that express PY03011 and PY03424, or PY03011, PY03424 and PY03661 protected mice at significantly higher levels than vaccines that express the individual antigens. Other studies have also shown that combining vaccines can enhance protection, as well as circumvent the HLA-restricted protection observed with some single antigen vaccines. For example, a combination vaccine containing two DNA vectors that express PyCSP and PyHEP17 protected a higher percentage of BALB/c, A/J and B10.BR mice than either the DNA-PyCSP or DNA-PyHEP17 vector alone [37]. In addition, monkeys immunized with DNA and vaccinia vectors expressing four *P. knowlesi *antigens (PkCSP, PkTRAP, PkAMA1 and PkMSP1_42_) controlled a *P. knowlesi *challenge significantly better than monkeys immunized with DNA and vaccinia vectors expressing only PkCSP [38]. Combining vaccines, however, can have several disadvantages. A multi-component vaccine may be more expensive to manufacture than a vaccine that contains a single component. In addition, there is a risk that one vaccine component can have an immunosuppressive effect on the other components. For example, a vaccine containing nine different DNA-*P. falciparum *vectors elicited significantly lower immune responses against each individual antigen than a vaccine containing the individual vectors [39]. Therefore, combining vaccine antigens will need to be evaluated empirically to see if synergistic, additive or antagonistic responses are observed.

## Conclusions

The results presented here suggest that characterizing the protective potential of new malaria vaccine antigens, such as PY03011 and PY03424, may contribute to the development of an efficacious malaria vaccine that can overcome the antigenic diversity of malaria parasites. In future studies, these antigens will be tested in combination with other protective antigens, such as PyCSP, to see if even higher levels of protection can be achieved.

## Competing interests

The authors declare that they have no competing interests.

## Authors' contributions

KL conceived and designed the experiments. KL, JA, KG, EA and JS performed the experiments. KL and NP analyzed the data. JA and MS contributed information and reagents. KL, NP and TR wrote the manuscript. All authors have read and approved the final manuscript.

## References

[B1] WHO World Malaria Report 2009 2009 Geneva: WHO Press, World Health Organization

[B2] Mita T Tanabe K Kita K Spread and evolution of *Plasmodium falciparum *drug resistance Parasitol Int 2009 58 201 209 10.1016/j.parint.2009.04.004 19393762

[B3] Hoffman SL Goh LM Luke TC Schneider I Le TP Doolan DL Sacci J de la Vega P Dowler M Paul C Gordon D Stoute J Church L Sedegah M Heppner D Ballou W Richie T Protection of humans against malaria by immunization with radiation-attenuated *Plasmodium falciparum *sporozoites J Infect Dis 2002 185 1155 1164 10.1086/339409 11930326

[B4] Vaughan A Wang R Kappe S Genetically engineered, attenuated whole-cell vaccine approaches for malaria Hum Vaccines 2010 6 107 113 10.4161/hv.6.1.9654 PMC3641786 19838068

[B5] Stoute JA Slaoui M Heppner DG Momin P Kester KE Desmons P Wellde B Garcon N Krzych U Marchand M A preliminary evaluation of a recombinant circumsporozoite protein vaccine against *Plasmodium falciparum *malaria. RTS,S Malaria Vaccine Evaluation Group N Engl J Med 1997 336 86 91 10.1056/NEJM199701093360202 8988885

[B6] Ballou WR The development of the RTS,S malaria vaccine candidate: challenges and lessons Parasite Immunol 2009 31 492 500 10.1111/j.1365-3024.2009.01143.x 19691554

[B7] Ewer K Collins K O'Hara G Duncan C Rowland R Reyes-Sandoval A Goodman A Poulton I Hutchings C Bird P Berrie E Nicosia A Colloca S Cortese R Siani L Lawrie A Gilbert S Hill A Protection from malaria sporozoite challenge correlates with frequency of TRAP-specific CD8+ T cells secreting IFNγ [abstract] Malaria: New Approaches to Understanding Host-Parasite Interactions 2010 s86

[B8] Chuang I Sedegah M Cicatelli S Spring M Tamminga C Bennett J Guerrero M Polhemus M Cummings J Angov E Bruder J Patterson N Limbach K Murphy J Bergmann-Leitner E Soisson S Diggs C Ockenhouse C Richie T Phase 1/2a Clinical Trial on Safety, Tolerability, Immunogenicity and Efficacy of Prime Boost Regimen of DNA- and Adenovirus-vectored Malaria Vaccines Encoding *Plasmodium falciparum *Circumsporozoite Protein (CSP) and Apical Membrane Antigen (AMA1) in Malaria-Naïve Adults [abstract] Malaria: New Approaches to Understanding Host-Parasite Interactions 2010 s83

[B9] Ogutu BR Apollo OJ McKinney D Okoth W Siangla J Dubovsky F Tucker K Waitumbi J Diggs C Wittes J Malkin E Leach A Soisson L Milman J Otien L Holland C Polhemus M Remich S Ockenhouse C Cohen J Ballou W Martin S Angov E Stewart V Lyon J Heppner D Withers M for the MSP-1 Malaria Vaccine Working Group Blood stage malaria vaccine eliciting high antigen-specific antibody concentrations confers no protection to young children in Western Kenya PLoS One 2009 4 e4708 10.1371/journal.pone.0004708 19262754 PMC2650803

[B10] Sagara I Ellis RD Dicko A Niambele MB Kamate B Guindo O Kante O Niambele M Miura K Mullen G Pierce M Martin L Dolo A Diallo D Doumbo O Miller L Saul A A randomized and controlled Phase 1 study of the safety and immunogenicity of the AMA1-C1/Alhydrogel + CPG 7909 vaccine for *Plasmodium falciparum *malaria in semi-immune Malian adults Vaccine 2009 27 7292 7298 10.1016/j.vaccine.2009.10.087 19874925 PMC2808270

[B11] Genton B Betuela I Felger I Al-Yaman F Anders RF Saul A Rare L Baisor M Lorry K Brown G Pye D Irving D Smith T Beck H Alpers M A recombinant blood-stage malaria vaccine reduces *Plasmodium falciparum *density and exerts selective pressure on parasite populations in a phase 1-2b trial in Papua New Guinea J Infect Dis 2002 185 820 827 10.1086/339342 11920300

[B12] Ouattara A Takala S Coulibaly D Amadou N Saye R Thera M Plowe C Doumbo O Allele-specific efficacy of an AMA-1-based malaria subunit vaccine [abstract] Am J Trop Med Hyg 2009 81 s162

[B13] Mikolajczak S Jacobs-Lorena V MacKellar D Camargo N Kappe S L-FABP is a critical host factor for successful malaria liver stage development Inter J Parasitol 2007 37 483 489 10.1016/j.ijpara.2007.01.002 17303141

[B14] Pandey KC Singh N Arastu-Kapur S Bogyo M Rosenthal PJ Falstatin, a cysteine protease inhibitor of *Plasmodium falciparum*, facilitates erythrocyte invasion PLoS Pathog 2006 2 1031 1041 10.1371/journal.ppat.0020117 PMC1630708 17083274

[B15] Florens L Washburn M Raine J Anthony R Grainger M Haynes J Moch J Muster N Sacci J Tabb D Witney A Wolters D Wu Y Gardner M Holder A Sinden R Yates J Carucci D A proteomic view of the *Plasmodium falciparum *life cycle Nature 2002 419 520 526 10.1038/nature01107 12368866

[B16] LeRoch D Zhou Y Blair P Grainger M Moch J Haynes J De La Vega P Holder A Batalov S Carucci D Winzeler Discovery of gene function by expression profiling of the malaria parasite life cycle Science 2003 301 1503 1508 10.1126/science.1087025 12893887

[B17] Sacci J Ribeiro J Huang F Alam U Russell J Blair P Witney A Carucci D Azad A Aguiar J Transcriptional analysis of *in vivo Plasmodium yoelii *liver stage gene expression Mol Biochem Parasitol 2005 142 177 183 10.1016/j.molbiopara.2005.03.018 15876462

[B18] Aguiar J LaBaer J Blair P Shamailova V Koundinya M Russell J Huang F Mar W Anthony R Witney A Caruana S Brizuela L Sacci J Hoffman S Carucci D High-throughput generation of *P. falciparum *functional molecules by recombinational cloning Genome Res 2004 14 2076 2082 10.1101/gr.2416604 15489329 PMC528923

[B19] Aguiar J Bolton J Wanga J Urquhart A Sacci J Limbach K Tsuboi T Ockenhouse C Richie T Discovering novel pre-erythrocytic antigens for malaria [abstract] Am J Trop Med Hyg 2009 81 s290

[B20] Perkus ME Limbach K Paoletti E Cloning and expression of foreign genes in vaccinia virus, using a host range selection system J Virol 1989 63 3829 3836 2547999 10.1128/jvi.63.9.3829-3836.1989 PMC250976

[B21] Chakrabarti S Sisler JR Moss B Compact, synthetic, vaccinia virus early/late promoter for protein expression Biotechniques 1997 23 1094 1097 9421642 10.2144/97236st07

[B22] Piccini A Perkus ME Paoletti E Vaccinia virus as an expression vector Methods Enzymol 1987 153 545 563 full_text 2828850 10.1016/0076-6879(87)53077-4

[B23] Tsuboi T Takeo S Arumugam TU Otsuki H Torii M The wheat germ cell-free protein synthesis system: A key tool for novel malaria vaccine candidate discovery Acta Trop 2010 114 171 176 10.1016/j.actatropica.2009.10.024 19913490

[B24] Charoenvit Y Leef MF Yuan LF Sedegah M Beaudoin RL Characterization of *Plasmodium yoelii *monoclonal antibodies directed against stage-specific sporozoite antigens Infect Immun 1987 55 604 608 2434426 10.1128/iai.55.3.604-608.1987 PMC260381

[B25] Sacci JB Jr Alam U Douglas D Lewis J Tyrrell DL Azad AF Kneteman N *Plasmodium falciparum *infection and exoerythrocytic development in mice with chimeric human livers Int J Parasitol 2006 36 353 360 10.1016/j.ijpara.2005.10.014 16442544

[B26] Wolff JA Malone RW Williams P Chong W Acsadi G Jani A Felgner P Direct gene transfer into mouse muscle *in vivo* Science 1990 247 1465 1468 10.1126/science.1690918 1690918

[B27] Sedegah M Weiss W Sacci JB Jr Charoenvit Y Hedstrom R Gowda K Majam V Tine J Kumar S Hobart P Hoffman S Improving protective immunity induced by DNA-based immunization: priming with antigen and GM-CSF-encoding plasmid DNA and boosting with antigen-expressing recombinant poxvirus J Immunol 2000 164 5905 5912 10820272 10.4049/jimmunol.164.11.5905

[B28] Sedegah M Charoenvit Y Aguiar J Sacci J Hedstrom R Kumar S Belmonte A Lanar D Jones T Abot E Druilhe P Corradon G Epstein J Richie T Carucci D Hoffman S Effect on antibody and T-cell responses of mixing five GMP-produced DNA plasmids and administration with plasmid expressing GM-CSF Genes Immun 2004 5 553 561 10.1038/sj.gene.6364125 15318164

[B29] PlasmoDB database http://PlasmoDB.org

[B30] Vaughan A Chiu SY Ramasamy G Li L Gardner MJ Tarun AS Kappe S Peng X Assessment and improvement of the *Plasmodium yoelii yoelii *genome annotation through comparative analysis Bioinformatics 2008 24 i383 389 10.1093/bioinformatics/btn140 18586738 PMC2718618

[B31] Matuschewski K Ross J Brown S Kaiser K Nussenzweig V Kappe S Infectivity-associated changes in the transcriptional repertoire of the malaria parasite sporozoite stage J Biol Chem 2002 277 41948 41953 10.1074/jbc.M207315200 12177071

[B32] Kaiser K Matuschewski K Camargo N Ross J Kappe SH Differential transcriptome profiling identifies *Plasmodium *genes encoding pre-erythrocytic stage-specific proteins Mol Microbiol 2004 51 1221 1232 10.1046/j.1365-2958.2003.03909.x 14982620

[B33] Spielmann T Fergusen DJ Beck HP etramps, a new *Plasmodium falciparum *gene family coding for developmentally regulated and highly charged membrane proteins located at the parasite-host cell interface Mol Biol Cell 2003 14 1529 1544 10.1091/mbc.E02-04-0240 12686607 PMC153120

[B34] Vignali M McKinlay A LaCount D Chettier R Bell B Sahasrabudhe S Hughes R Fields S Interaction of an atypical *Plasmodium falciparum *ETRAMP with human apolipoproteins Malar J 2008 7 211 10.1186/1475-2875-7-211 18937849 PMC2577112

[B35] Tarun AS Dumpit RF Camargo N Labaied M Liu P Takagi A Wang R Kappe S Protracted sterile protection with *Plasmodium yoelii *pre-erythrocytic genetically attenuated parasite malaria vaccines is independent of significant liver-stage persistence and is mediated by CD8+ T cells J Infect Dis 2007 196 608 616 10.1086/519742 17624848

[B36] Rennenberg A Lehmann C Heitmann A Witt T Hansen G Nagarajan K Deschermeier C Turk V Hilgenfeld R Heussler V Exoerythrocytic *Plasmodium *parasites secrete a cysteine protease inhibitor involved in sporozoite invasion and capable of blocking cell death of host hepatocytes PLoS Pathog 2010 6 e1000825 10.1371/journal.ppat.1000825 20361051 PMC2845656

[B37] Doolan DL Sedegah M Hedstrom RC Hobart P Charoenvit Y Hoffman SL Circumventing genetic restriction of protection against malaria with multigene DNA immunization: CD8+ cell-, interferon gamma-, and nitric oxide-dependent immunity J Exp Med 1996 183 1739 1746 10.1084/jem.183.4.1739 8666931 PMC2192484

[B38] Weiss WR Kumar A Jiang G Williams J Bostick A Conteh S Fryauff D Aguiar J Singh M O'Hagan D Ulmer J Richie T Protection of rhesus monkeys by a DNA prime/poxvirus boost malaria vaccine depends on optimal DNA priming and inclusion of blood stage antigens PLoS One 2007 2 e1063 10.1371/journal.pone.0001063 17957247 PMC2031826

[B39] Sedegah M Charoenvit Y Minh L Belmonte M Majam VF Abot S Ganeshan H Kumar S Bacon D Stowers A Narum D Carucci D Rogers W Reduced immunogenicity of DNA vaccine plasmids in mixtures Gene Ther 2004 11 448 456 10.1038/sj.gt.3302139 14973538

